# Alcohol Consumption in the Specific Socio-Professional Context of the French Public Service: Qualitative Study Protocol

**DOI:** 10.3390/ijerph192315915

**Published:** 2022-11-29

**Authors:** Benjamin du Sartz de Vigneulles, Florence Carrouel, Elise Verot, Christian Michel, Thierry Barthelme, Jean-Charles Pere, Roger Salamon, Claude Dussart

**Affiliations:** 1 Laboratory “Health, Systemic, Process” (P2S), UR4129, University Claude Bernard Lyon 1, University of Lyon, 69008 Lyon, France; 2 University Jean Monnet, 42270 Saint-Etienne, France; 3 Equipe PREDUCAN, CIC Inserm 1408 Saint-Etienne, 42055 Saint-Etienne, France; 4 Practice for Addiction Medicine-Association for Prevention and Rehabilitation (gGmbH), 77694 Kehl, Germany; 5 French Society of Officinal Pharmaceutical Sciences (SFSPO), 91570 Bièvres, France; 6 Institute of Public Health, Epidemiology and Development (ISPED), Inserm U1219, University of Bordeaux, 33000 Bordeaux, France; 7 Hospices Civils de Lyon, 69002 Lyon, France

**Keywords:** alcohol, addiction, misuse, consumption, workplace, public service, psychoactive substance, focus group, disorder

## Abstract

Alcohol, a psychoactive substance with addictive potential, has major consequences on the population and public health. In France, alcohol use disorder affects approximately 3.5 million people, and 41,000 persons died in 2015. Alcohol consumption is significantly correlated to the workplace. Thus, the workplace is an area of opportunity to change risky behaviors and must play a key role in the prevention of alcohol misuse. To do this, it is essential to understand the consumption framework and to identify specific environmental risk factors. This qualitative study aims to describe the framework of alcohol consumption in the French public service. A focus group will be organized in France from November to January 2023. The participants will be: (i) representatives of the Local Health Insurance; (ii) over 18 years old; (iii) active or retired civil servants; (iv) mutualist activists; and (v) representatives of the Union of Health Prevention for the Obligatory System of the Public Service. The exclusion criteria for the study will be: (i) lack of consent form; (ii) inability to participate in the focus group, and (iii) early departure during the focus group. The focus groups will be supervised by two researchers following an interview guide. The data will be analyzed using the methodological framework, which consists in carrying out a thematic analysis. This will allow for an understanding of the sources of usage behaviors, and the identification of the most appropriate intervention functions for suitable prevention actions in order to reduce the risk of a transition to alcohol use disorder.

## 1. Introduction

Alcohol is generally considered as the only psychoactive substance with addictive potential “that is not controlled at the international level by legally binding regulatory frameworks”, even though it has major population and public health consequences [1]. Three million deaths per year and more than two hundred diseases and injuries (domestic accidents, road traffic, liver cirrhosis, cancer, infectious diseases, alcoholic cardiomyopathy, stroke…) are associated with the consumption of alcohol [1]. Overall, 5.1% of the global burden of disease and injury is attributable to alcohol, measured in disability-adjusted life years (DALYs) [1]. Mortality due to alcohol consumption is higher than that caused by diseases such as tuberculosis, HIV/AIDS, and diabetes [1].

In addition, alcohol consumption also represents a significant economic burden [2]. Its weighted average cost was 2.5% of gross domestic product (GDP) in purchasing power parity (PPP) terms in high-income countries (such as the United States, Canada, Scotland and France) [3]. This economic burden will increase in low and middle-income countries as per capita alcohol consumption in these countries increases with GDP-PPP [4]. However, the Organization for Economic Co-operation and Development (OECD) has shown that the implementation of policies to address heavy drinking can reduce regular and episodic alcohol consumption and alcohol dependence by up to 5–10% [5]. Indeed, even the most expensive alcohol policies have favorable cost-effectiveness profiles. For example, the World Health Organization (WHO) has shown that brief advice from physicians or increased taxes on alcohol have incremental cost-effectiveness ratios well below one per GDP per capita for many countries on all continents [6].

Despite a reduction of drinkers worldwide of about 5% from 47.6% to 43.0% since 2000, alcohol is still consumed by more than half of the population in three WHO regions, which include the European Region [1].

Alcohol affects not only personal life but also work life. Indeed, higher levels of alcohol consumption are associated with higher levels of impaired performance at work. In the workforce, alcohol is the most widely used psychoactive substance, but also the most misused [7]. Thus, 1 to 3 in 10 employees may be considered at-risk drinkers in need of intervention [8,9], i.e., with a pattern of use that increases the risk of social, legal, medical, occupational, domestic, and economic problems [8]. Alcohol decreases employment and increases unemployment, absenteeism and negatively influence productivity and work performance [10]. On the contrary, workers who had positive and/or grateful feedback on their work or were considered to have time to do their job well or have the freedom to organize their work had the lowest “Fast Alcohol Consumption Evaluation” score [11]. Several studies have demonstrated the socio-professional status impact on alcohol use disorder [12,13,14,15,16].

Thus, the workplace can be seen as a living environment where specific structural and social determinants of health intersect [17], particularly with regard to alcohol consumption. The workplace could influence workers and those who do not drink in three ways: (i) through workplace alcohol beliefs (physical availability of alcohol, ease of obtaining and consuming it during work hours and breaks); (ii) through descriptive norms (members of an individual’s social network drink or work under the influence of alcohol at work); and (iii) through injunctive norms (members of an individual’s social network approve of drinking or working under the influence of alcohol at work) [18]. So, as an environment that can be significantly correlated with alcohol consumption, the workplace is an area of opportunity to change risky behaviors in relation to individual capacities and personal motivation [19]. This is why the workplace must play a key role in prevention [20]. To do this, it is essential to understand the consumption framework, and to identify specific environmental risk factors which can negatively impact other characteristics of health behaviors and may lead to alcohol misuse.

In France, alcohol use disorder is a major public health problem affecting approximately 3.5 million people (7% of adults). In 2015, 41,000 persons died [21]. Less than half of French people who identified as having an alcohol use disorder used mental health care in the previous 12 months and, overall, about 10% of people with an alcohol use disorder receive related medical care [22].

Currently, there is a growing French political consideration of the use of psychoactive substances in the workplace, particularly in the public service [23,24,25]. It could be interesting to study the alcohol consumption in the specific socio-professional context of the French public service for several reasons. First, these public agents evolve in a specific work context, with a job guarantee, and relatively strong stability of their professional environment. Second, several socio-professional categories are represented [26]. Third, this population has few occupational physicians, and some organizations must fill this gap in the prevention network. Finally, the health management of public servants is unique in the French public health system, with management being more often delegated to the mutual system [27] for a more preventive orientation.

The objective of this study will be to describe, according to the opinions of specific experts (civil servants (active or retired) who are mutualist activists, and health prevention representatives for the Obligatory System of the Public Service), the framework of alcohol consumption in the French public service. Particularly, this study will focus on “alcohol and work” (typologies of alcohol consumption at work, perceived risks, specificities to the public service) and on “alcohol and mental suffering” (possible links between the mental state of officials and their alcohol consumption). This should allow for an understanding of the sources of drinking behavior and an identification of the most appropriate intervention functions for appropriate prevention measures, in order to reduce the risk of transition to an alcohol use disorder.

## 2. Materials and Methods

### 2.1. Study Design

This qualitative study will adopt a descriptive design focusing on the framework of alcohol use in the public service workplace. Data collection will be carried out through a focus group that will be organized in France from November to January 2023.

### 2.2. Selection of Participants

All the participants of this study will be representatives of the Local Health Insurance (*Section Locale d’Assurance Maladie*, SLAM). The SLAM representatives are civil servants (active or retired), mutualist activists, and are also representatives of the Union of Health Prevention for the Obligatory System of the Public Service (*Union de mutuelles pour la prévention santé du Régime obligatoire pour la Fonction publique, Urops*). They are in charge of setting up prevention actions in public administrations. Thus, they are very familiar with the professional social framework of the public service and, as “enlightened colleagues”, they could have a general, non-autocentric and relevant point of view on the subject of alcohol consumption in the public service and related use disorders. SLAM representatives will not talk about their personal alcohol use but will share their opinions in an attempt to define the place of alcohol in the public service.

To be included, participants should be: (i) SLAM representatives; (ii) over 18 years old; (iii) active or retired civil servants; (iv) mutualist activists; and (v) Urops representatives.

Exclusion criteria for the study will be: (i) lack of consent form; (ii) inability to participate in the focus group; and (iii) early departure during the focus group.

### 2.3. Sampling

As recommended for focus group research, the strategy will be to select purposive sampling whereby the researcher will select participants [28] in a way that will provide an understanding of alcohol problems in the public service. Thus, in order to obtain rich information and to achieve maximum participant variation sampling, we will enroll SLAM representatives who: (i) come from different regions (homogeneous distribution across France); (ii) are related to different socio-professional categories in the civil service; and (iii) are very interested in the study of alcohol addiction and willing to discuss their perspectives on our research topic.

Recruitment will be carried out by sending an email to the SLAM representatives in France. This message will include a recruitment announcement, a study information sheet, and a consent document. Individuals interested in participating will be asked to indicate their intention by sending an e-mail to the person in charge of this research. In the e-mail response, the participant will be asked to specify: gender; employment status (working or retired); age range (according to scale); occupation or former occupation; category of civil servant; number of years as a civil servant; number of years as a SLAM representative; and the size of the city of their last employment. This will ensure a balanced distribution of the sample. This will also make it possible to generate an anonymous identification code (which will be sent to the participant by return e-mail, along with the interview appointment information). This individual and anonymous ID code will first allow the focus group animator to have a list of participants. This code will then be used to link these socio-demographic data to the stories that will be told during the interviews (a desk sign bearing this ID code will be placed in front of the participant in the meeting room). Later, during the analysis phase, cross-referencing these data will eventually allow us to find explanatory elements for the points of view.

### 2.4. Sample Size

In qualitative research, the number of interviewees is not a determining factor in the significance of the results. Data saturation through redundancy will be the main indicator. Thus, an initial sample size is estimated but the final sample size will be determined by data saturation [29].

Concerning this specific population of SLAM representatives, in the absence of previous knowledge about their participation in focus groups, it is difficult to estimate precisely the number of participants. There is one SLAM per geographic department and five and 12 representatives in a SLAM, that is to say about 500 SLAM representatives in France. They are mostly from the state civil service (excluding national education), but within the state civil service, they cover a wide variety of professions and all categories of agents.

Given that it is recognized that, in the case of a homogeneous sample, (i) the first five–six participants produce the majority of new information in the dataset, while little information is obtained from subsequent participants, and (ii) that data from the first 10 participants identify 80–92% of the information [30], and that when the sample size is around 20 interviews then little new information will be collected [31], the five focus groups consisting of six to 12 SLAM representatives (30 to 60 participants) will be organized to allow for r data saturation (i.e., when no new codes or themes emerge). Focus group interviews will be discontinued when data saturation is reached.

### 2.5. Development of the Interview Guide

The guide could not be developed on models pre-used or validated in previous studies because an analysis of the literature did not reveal any. It was therefore designed by the research team on the basis of expert consensus. The experts are specialists in public health, human and social sciences and addictology. This guide will be tested during three individual interviews, by telephone, with SLAM representatives who will not participate in our study. These interviews will not be recorded, and will not be included in future results, but will allow us to collect suggestions on the questions and adapt the guide. The questions included in the interview guide are described in Table 1.

The interview guide is structured in three parts according to a progression corresponding to the subject of the research.

The first part deals with the theme of alcohol and work. Its objectives are to explore the perceived link between alcohol consumption and the professional social framework, to highlight typologies of alcohol consumption at work, to identify the perceived risks (and to understand the trivialization and/or or denial), and possibly to notice specificities in the public service. Composed of nine exclusively open-ended questions, it begins with “What does the word “alcohol” mean to you? », and ends with « What do you think of the trivialization of the regular consumption of alcohol?”.

The second part deals with the theme of alcohol and psychological suffering. Its objectives are to analyze the perceived link between alcohol consumption and mental health, and to know the mental state of civil servants, and its impact on alcohol consumption. It is composed of three open-ended questions, such as “In general, how is the morale of civil servants?”, and ends voluntarily with a semi-closed question: “Do you have examples, without naming them, of colleagues who associate regular alcohol consumption and psychological suffering?”.

The third part aims to address axes of solutions. In addition to providing food for thought, it can help to reformulate the problem. It is made up of a single question: “Finally, what could you suggest to reduce the alcohol consumption of civil servants?”.

### 2.6. Description of the Interview and Data Collection

The focus groups will be supervised by two researchers: C.M. who is a doctor, addictologist, doctor in human and social sciences, and B.D. who is a doctoral student trained in qualitative research.

For ethical reasons and respect for confidentiality, these two researchers will not know the participants (only the research manager will be aware of personal data). They will only know the number and the breakdown by gender of the group.

Upon arrival, the participant will sit down in the group interview room where the desk sign with his or her ID code is located. The interviews will be filmed in order to link these socio-demographic criteria (by vision of the sign) to the remarks made: this will be done in the written transcription by affixing the identifying code before the verbatim, and this will make it possible to better contextualize the story. The video recording will be deleted after this operation is completed. In parallel, a correspondence table will present the link between identifying code and socio-demographic criteria. This data will remain within the research team.

The five focus groups will be led by the same person (C.M.), who is used to this kind of interview as the e second team member (B.D.) will be present as an observer/moderator. The interview will begin with the presentation of the facilitator and a reminder of the purpose of the meeting. Time for discussion on this study and to answer any questions will be offered.

The guide will be used as little as possible, only in case of necessity, in order to obtain spontaneous answers and limit the risk of orientation of the respondents’ speech. Reminders or requests for reformulation will be proposed as a priority to develop the exploration.

After each focus group, an immediate “live” assessment will be carried out between the two researchers present, then a subsequent “cold” report will be carried out with the rest of the research team. The main points will be recorded.

### 2.7. Data Analysis

To analyze these qualitative data, the methodological framework proposed by Braun and Clarke (2006) [32], which consists in carrying out a thematic analysis, will be applied. The recordings of the speeches will be transcribed verbatim, following the exact course of the interview, and mentioning all the elements of communication, including the non-verbal cues, in order to fully understand the interactions (laughs, hesitations, attitudes). Then, the verbatims will be analyzed using NVivo 11 pro software (QSR International, Burlington, MA, USA). Coding and thematic analysis will be carried out by the researchers who carried out the interviews. The report of the qualitative analysis will follow the COREQ (COnsolidated criteria for REporting Qualitative research), guidelines recommended by the EQUATOR network (Enhancing the QUAlity and Transparency Of health Research) for reporting qualitative research. The results will then be discussed under the lens of the theoretical frameworks mobilized within the various disciplines making up the research team. The results will also be compared with the literature.

At the end of the analysis, two or three individual non-directive interviews with study participants will be conducted to present the emerging concepts and discuss their coherence in order to corroborate the results obtained.

### 2.8. Ethical Approval

Ethical approval was obtained from the Research Ethics Committee of the University of Lyon (n°2022-09-15-004 on 18 October 2022) and a declaration (MR004) to the National Commission for Computing and Liberties (CNIL) (2226244 v 0 was made on 5 May 2022. Written informed consent will be obtained from eligible participants before data collection.

## 3. Discussion

Exploring the consumption of alcohol in the specific socio-professional context of the French public service is a significant topic due to the lack of relevant knowledge. This study will be the first study analyzing the consumption of alcohol in the specific socio-professional context of the French public service. Due to the fact that alcohol consumption has increased in the French public service following the COVID-19 crisis [33], it is likely that the pandemic variable will be an inherent aspect of the investigation. Thus, this study will identify explanatory elements for the increase in alcohol consumption in the French public service following the COVID-19 crisis, its banalization, and the link with personal and professional psychological suffering. Thus, it is possible that the data collected during the focus groups will permit a classification of alcohol use disorders in the public service based on the fifth edition of the Diagnostic and Statistical Manual of Mental Disorders and Psychiatric Disorders (DSM-5) of the American Psychiatric Association [34]. This knowledge of the typologies of alcohol use (mild, moderate or severe alcohol use disorders) in the professional social setting will make it possible, according to the alcohol health-related risk and treatment response pyramid, to formulate more appropriate prevention measures [35].

This study has several strenghs. First, the choice of interviewing SLAM representatives, who may be witnesses to alcohol-related problems without being directly involved because people affected by alcohol problems find it difficult to talk about them. Second, even if some SLAM representatives are retired, they have a perfect knowledge of health problems in the workplace because they are in charge of carrying out prevention/health promotion actions in the public service workplaces. Third, the choice of focus-group interviews that involve group interactions will generate deeper and richer data in many scenarios [36]. Focus group interviews collect data from each participant and generate new questions and answers through interactive verbal communication among group members. This provides an opportunity to learn about the needs and feelings of participants and to explore the influence of cultural values and beliefs [28].

This study has several limitations. First, due to the fact that SLAM representatives do not have clinical expertise in mental health, the data collected will be personal perceptions of a social environment but not a diagnosis. Second, the choice of the participant can lead to “confirmation bias” because humans tend to select information that is consistent with what they believe (or want to believe) and interpret the information available to them in favor of their preferred hypotheses [37]. Third, the interview guide was designed for this study and is not based on previously published research. It will be tested for validation. Fifth, since SLAM representatives are scattered throughout France, a face-to-face meeting will be held at a geographic point that will require some participants to change departments, which can be complicated, and this can inhibit the desire to participate.

## 4. Conclusions

The findings of this qualitative study could help to develop and improve specific programs to prevent the consumption of alcohol among public service employees. Prevention programs could be implemented by mutual organizations that have become important actors in prevention actions by delegation from the public authorities. In addition, a French civil servant is covered by a mutual insurance company (usually the same one) from his first day of work until his death, i.e., when he is active and then retired. Thus, the insurees could be studied longitudinally to highlight the impact of selective prevention on behaviors regarding the consumption of alcohol.

## Figures and Tables

**Table 1:** Interview guide.

Topics	Objectives	Questions
Alcohol and work	Explore the perceived relationship between alcohol use and the occupational social setting - Identify typologies of alcohol use in the workplace. - Identify perceived risks (/trivialization, denial). - Determine if there are any specificities in the public service.	- What does the word “alcohol” mean to you? - What link(s) can there be between alcohol and work? - Why does the law allow for the (controlled) consumption of alcohol in the workplace? - What are the potential risks associated with alcohol consumption in your work environment? - How is the notion of “alcohol abuse” perceived in the public service? - What are the differences between the public and private sectors regarding alcohol use? - What are some examples you can cite of alcohol consumption in the workplace? - What do you think about alcohol consumption and the transition to retirement? - What do you think about the trivialization of regular drinking?
Alcohol and psychic suffering	Analyze the perceived relationship between alcohol use and mental health. - To know the psychological state of the public workers, and its impact on the consumption of alcohol.	- In general, how is the morale of public workers? - How has the morale of public workers changed in recent years? - What link(s) might there be between alcohol consumption and mental state? - Do you have examples, without naming them, of colleagues who associate regular alcohol consumption with psychological suffering?
Potential solutions	Suggesting solutions to improve the situation, in addition to providing points for discussion, can help to reformulate the problem.	- Finally, what would you suggest to reduce alcohol consumption by public workers?

## Data Availability

Not applicable.
